# A prospective, randomized, controlled trial of TB treatment shortening guided by PET/CT imaging

**DOI:** 10.1126/scitranslmed.adt5626

**Published:** 2026-01-07

**Authors:** Stephanus T. Malherbe, Ray Y. Chen, Xiang Yu, Bronwyn Smith, Xin Liu, Jingcai Gao, Andreas H. Diacon, Rodney Dawson, Michele Tameris, Hong Zhu, Yahong Qu, Hongjian Jin, Shouguo Pan, Lori E. Dodd, Jing Wang, Lisa C. Goldfeder, Ying Cai, Kriti Arora, Joel Vincent, Kim Narunsky, Keboile Serole, Rene T. Goliath, Laylah Da Costa, Arshad Taliep, Saalikha Aziz, Remy Daroowala, Friedrich Thienemann, Sandra Mukasa, Richard Court, Bianca Sossen, Petri Ahlers, Simon C. Mendelsohn, Lisa White, Aurélie Gouel, Chuen-Yen Lau, Samy Hassan, Lili Liang, Hongfei Duan, Gita K. Moghaddam, Praveen Paripati, Saher Lahouar, Michael Harris, Kurt Wollenberg, Brendan Jeffrey, Mike Tartakovsky, Alex Rosenthal, Michael Duvenhage, Derek T. Armstrong, Taeksun Song, Jill Winter, Qian Gao, Laura E. Via, Robert J. Wilkinson, Gerhard Walzl, Clifton E. Barry

**Affiliations:** 1 DST-NRF Centre of Excellence for Biomedical Tuberculosis Research, South African Medical Research Council Centre for Tuberculosis Research, Division of Immunology, Department of Biomedical Sciences, Faculty of Medicine and Health Sciences, Stellenbosch University, Cape Town, South Africa; 2 Tuberculosis Research Section, Laboratory of Clinical Immunology and Microbiology, Division of Intramural Research, National Institute of Allergy and Infectious Disease, National Institutes of Health, Bethesda, MD, USA; 3 Henan Provincial Chest Hospital, Zhengzhou, Henan, China; 4 Sino-US Tuberculosis Collaborative Research Program, Zhengzhou, Henan, China; 5 TASK, Cape Town, South Africa; 6 Division of Pulmonology, Department of Medicine, University of Cape Town Lung Institute, Centre for TB Research Innovation, Observatory 7923, Republic of South Africa; 7 South African Tuberculosis Vaccine Initiative, Institute of Infectious Disease and Molecular Medicine, Department of Pathology, University of Cape Town, Observatory 7923, Republic of South Africa; 8 Kaifeng City Institute of Tuberculosis Prevention and Control, Kaifeng, Henan, China; 9 Xinmi Center for Disease Control and Prevention, Xinmi, Henan, China; 10 Zhongmu County Health and Epidemic Prevention Station, Zhongmu, Henan, China; 11 Clinical Clinical Trials Research and Statistics Branch, Office of Biostatistics Research, Division of Clinical Research, National Institute of Allergy and Infectious Disease, National Institutes of Health, Bethesda, MD, USA; 12 Clinical Monitoring Research Program Directorate, Frederick National Laboratory for Cancer Research, Frederick, MD, USA; 13 University of Cape Town Lung Institute (Pty) Ltd, Mowbray, Cape Town, 7700, Republic of South Africa; 14 Centre for Infectious Diseases Research in Africa, Institute of Infectious Disease and Molecular Medicine, University of Cape Town, Observatory 7925, Republic of South Africa; 15 Department of Internal Medicine, University Hospital Zurich, University of Zurich, Zurich, Switzerland; 16 Department of Medicine, University of Cape Town, Observatory 7923, Republic of South Africa; 17 HIV Dynamics and Replication Program, National Cancer Institute, National Institutes of Health, Bethesda, MD, USA; 18 Beijing Chest Hospital, Beijing, China; 19 Department of Clinical Neurosciences, University of Cambridge, UK; 20 Office of Cyber Infrastructure and Computational Biology, NIAID, NIH, Bethesda, MD, USA; 21 Research Data and Communication Technologies, Inc., Garrett Park, MD, USA; 22 Department of Pathology, Johns Hopkins University, Baltimore, MD, USA; 23 Catalysis Foundation for Health, San Ramon, CA, USA; 24 School of Basic Medical Science, Shanghai Medical College, Fudan University, Shanghai, China; 25 Francis Crick Institute, London, NW1 1AT, UK; 26 Department of Infectious Diseases, Imperial College London, W12 0NN, UK

## Abstract

Six months of drug treatment is standard of care for drug-sensitive pulmonary tuberculosis (TB). Understanding the factors determining the length of treatment required for durable cure would allow individualization of treatment durations. We conducted a prospective, randomized, controlled noninferiority trial (PredictTB) of four versus six months of chemotherapy in patients with pulmonary TB in South Africa and China. 704 participants with newly diagnosed, drug-sensitive TB were enrolled and stratified based on radiographic disease characteristics assessed by FDG PET/CT imaging. Participants with less extensive disease (n=273) were randomly assigned at week 16 to complete therapy after four months or continue receiving treatment for six months. This study was stopped early after an interim analysis revealed that patients assigned to the four-month treatment arm had a higher risk of relapse. Amongst participants who received four months of therapy, 17 of 141 (12.1%) experienced TB-specific unfavorable outcomes compared to only 2 of 132 (1.5%) who completed six months of treatment. In the non-randomized arm that included participants with more extensive disease, only 8 of 248 (3.2%) experienced unfavorable outcomes. Total lung cavity volume and total lesion glycolysis at week 16 were associated with risk of unfavorable outcomes. Based on PET/CT imaging at TB recurrence, bacteriological relapses predominantly occurred in active cavities originally present at baseline. Subsequent post-hoc automated segmentation of serial PET/CT scans combined with machine learning enabled classification of participants according to their likelihood of relapse. These results may enable more efficient studies of TB treatment regimens in the future.

## Introduction

Treatment of drug-susceptible tuberculosis (TB) with the drugs currently in use typically requires at least six months of treatment to achieve durable cure (defined as failure to relapse after drug discontinuation) (1, 2). A four-month regimen is available but not yet widely used (2, 3). Patients with drug-susceptible TB receive an initial two month “intensive phase” with four drugs daily (isoniazid, rifampicin, pyrazinamide and ethambutol) followed by 4 months of therapy with two of these agents (isoniazid and rifampicin). Failure to convert sputum smear or culture by two months or the presence of cavities on chest X-rays at baseline are used in high resource countries as a basis for extending the two drug “continuation phase” by an additional three to nine full months. Many high TB burden countries follow the guidelines of the World Health Organization which stipulate 4-6 months of daily therapy for all TB patients (4).

The pioneering trials of the British Medical Research Council (BMRC) that led to the establishment of this standard of care regimen involved exploration of the duration of both the intensive and continuation phases and noted a curious phenomenon that about 80% of patients could be treated for only three months and be cured of their disease but to cure the remaining 20% the duration of therapy had to be extended for a full six months (5). This bimodal distribution of treatment responses suggests an underlying difference in the disease characteristics of patients cured early in treatment compared to those who require extended treatment times. One characteristic that could be different between these groups is the presence and extent of lung cavitary disease that has been associated with disease relapse in many studies (6–10). Both the presence of cavities and cavity size correlate with higher sputum bacterial load and disease severity (11–14). Another characteristic that is associated with risk of relapse is the susceptibility of individual TB strains to concentrations of rifampicin and isoniazidthat are not high enough to meet clinically established breakpoints to designate them as resistant but nonetheless potentially confer a survival benefit in some niches in patients (15).

Until the success of the TBTC Study 31/ACTG A5349 trial (16), no trial testing a 4-month treatment arm achieved non-inferiority compared to the 6-month standard of care; this included the older BMRC trials as well as the more recent fluoroquinolone-based trials (17–19). These trials enrolled participants without regard to disease severity, with the three fluoroquinolone-based studies having unfavorable outcomes ranging from 15-20% relapse in the 4-month arms. In contrast, Johnson et al. conducted a 4-month treatment shortening trial in which only participants with less severe disease, defined as no cavity on baseline chest x-ray and negative week 8 sputum culture, were randomized to 4 versus 6 months of treatment (20). This study revealed a 7% unfavorable outcome rate in the 4-month arm, driven largely by African participants, which was markedly worse than the 1.6% unfavorable outcome rate in the 6-month arm.

To identify the biological basis for the elevated risk of TB relapse in individual patients, we conducted a prospective, randomized controlled noninferiority trial of four versus six months of chemotherapy in patients with pulmonary TB using a combination of radiographic characteristics at baseline, the amount of change of these features at one month, and markers of residual bacterial load at the end of treatment. We selected radiological cutoffs based on treatment failures (n=8) and presumed relapses (n=11) from a smaller cohort of 95 individuals from South Africa treated for six months who had repeated PET/CT imaging scans and bacteriology (21). Although this was an imperfect comparator it was critical to know what lesions in which patients were responsible for the six-month duration of treatment to identify target parameters for new agents to achieve future treatment shortening.

## Results

### Study participants and randomization

**Figure 1:**
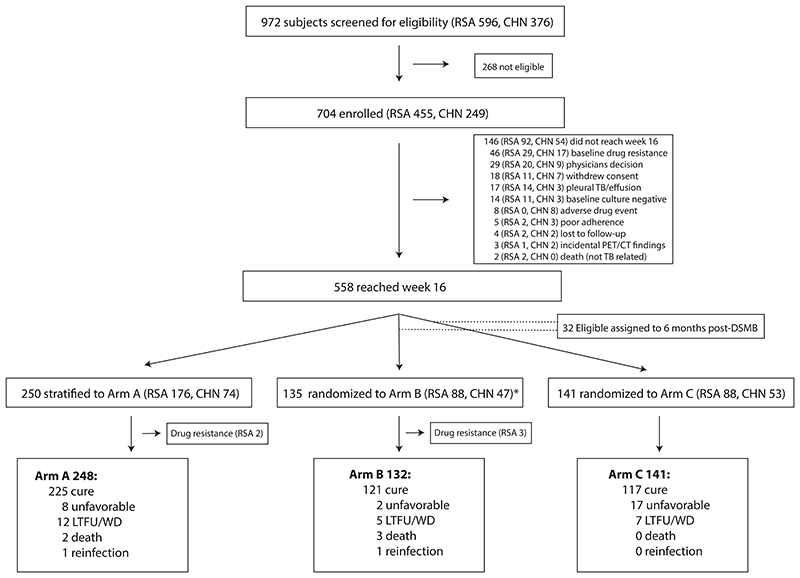
CONSORT flow diagram. Shown is the CONSORT flow diagram for patient recruitment in the PredictTB trial. 972 patients with newly diagnosed, drug sensitive TB were screened at multiple sites in South Africa (RSA) and China (CHN). 704 participants were enrolled and 146 were withdrawn from the study (reasons for ineligibility are presented in Table S1). Of the 558 participants included in the study, 248 were stratified to the non-randomized arm A due to disease severity and received the full six months of standard therapy, and 276 were randomly assigned to Arm B or Arm C where they were randomized at 4 months to either continue to receive treatment for an additional two months (Arm B) or discontinued at four months (Arm C). *After the DSMB stopped randomization, an additional 32 participants (11 in South Africa, 21 in China) in the Arm B/C pool were assigned to Arm B (not shown).

This was a prospective, randomized, controlled noninferiority trial of four versus six months of chemotherapy in patients with pulmonary TB in South Africa and China. We screened 972 participants with newly diagnosed, drug-sensitive pulmonary TB and enrolled a total of 704 of whom 558 were available for stratification or randomization at week 16 (Figure 1). Based on high baseline lung cavity and lesion burden, deterioration from baseline to week 4 (determined by repeat PET/CT imaging), or week 16 sputum GeneXpert MTB/RIF cycle threshold values ≥28, 250 participants (44%) were not eligible for randomization and were stratified to receive standard treatment for 24 weeks (Arm A). Two of these participants were subsequently found to have drug resistance and were withdrawn. 276 (49%) participants were eligible for randomization to receive either 24 weeks of treatment (Arm B, 135 participants, 3 of whom were subsequently found to have baseline drug resistance and were withdrawn from the study), or to stop treatment after 16 weeks (Arm C, 141 participants). During interim analysis, the Data Safety and Monitoring Board reviewed the results and stopped recruitment into the 16-week treatment arm due to meeting pre-specified stopping criteria for inferiority compared to 24-weeks of treatment. Thirty-two participants who were enrolled in the study at this time but not yet randomized and were not stratified to Arm A, were subsequently assigned to Arm B.

Baseline characteristics did not differ by study arm except for radiological features (cavities, hard CT volume, and total lesion glycolysis). Study participants assigned to Arm A, by design, had larger and more numerous cavities, larger CT hard volumes, greater total lesion glycolysis and higher bacterial burden (Table 1). By country, baseline characteristics were different in that Chinese participants had less extensive disease with fewer and smaller cavities, lower total lesion glycolysis, and lower bacterial burdens as assessed by higher GeneXpert cycle threshold (Ct), a PCR based assessment of bacterial DNA and longer Mycobacteria Growth Indicator Tube (MGIT) time to detection (TTD), a bacterial growth assay reporting days to a positive culture. Consistent with this, amongst participants assigned to Arm A, 70.2% were from South Africa and only 29.8% were Chinese. Participants were assigned to Arm A primarily because of baseline total lesion glycolysis exceeding 1500 followed by baseline hard volume greater than 200 mL and week 16 GeneXpert Ct values less than 28 (Figure S1).

### Study final outcomes

**Figure 2:**
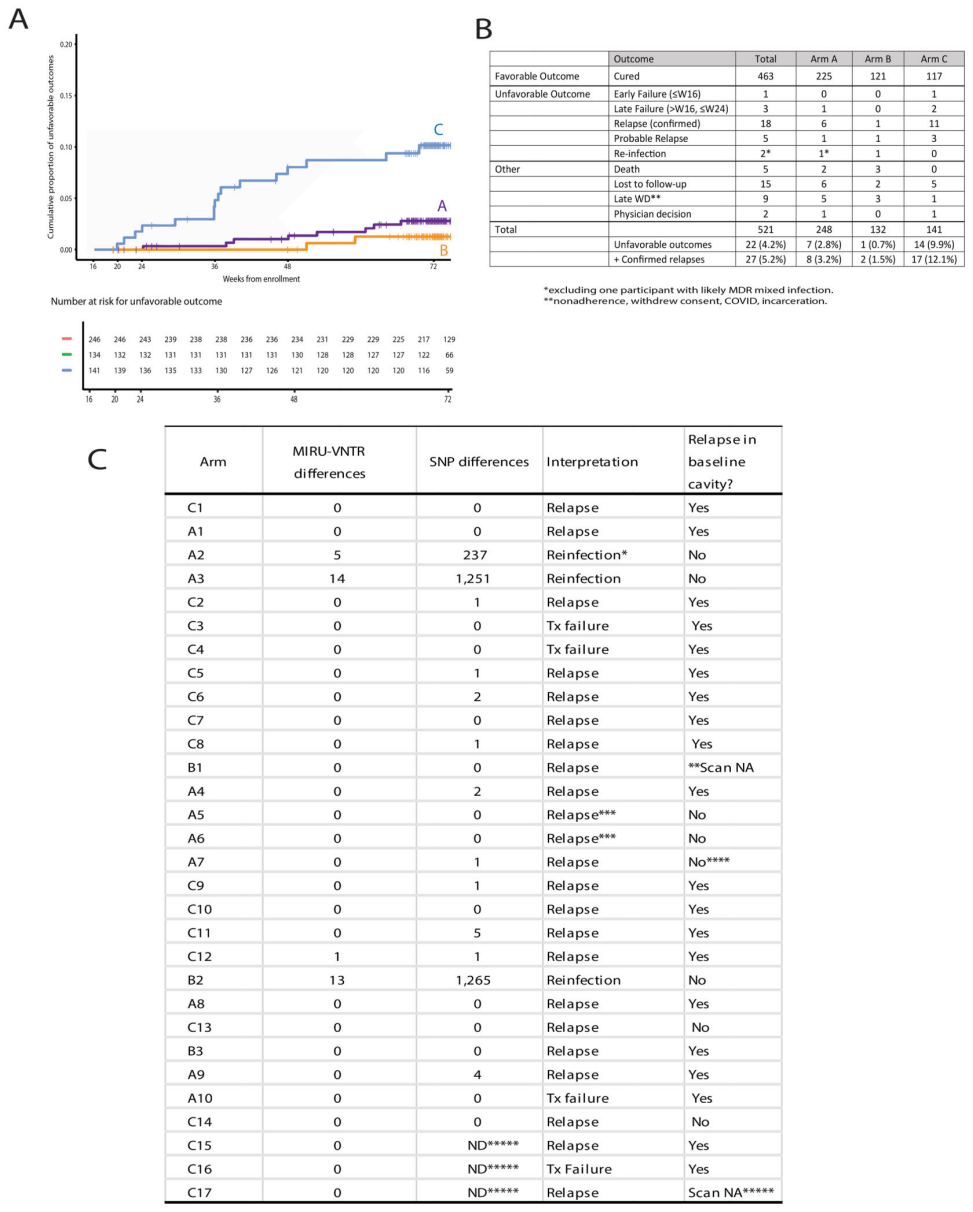
Final study outcomes. (**A)** Shown is a Kaplan-Meier plot presenting the cumulative proportion of unfavorable outcomes starting at week 16, the time of randomization. (**B**) Table indicates study participant final outcomes by Arm. (**C**) Table presents unfavorable outcomes with genomic information, interpretation and whether the relapse involved a lung cavity present at baseline. *The week 16 isolate was multi-drug resistant (MDR), which may have been from a mixed infection initially. **Participant had a stroke and was not able to undergo PET/CT imaging. ***A5 and A6were relatives and cohabitated during the study, re-infection with the same strain cannot be definitively ruled out (Figure S2). ****Relapse in lower lobe of the lung adjacent to the cavity that infiltrated the fissure. *****Whole genome sequencing data and final imaging scan were not available for these Chinese participants.

Overall treatment outcomes at the end of the study were assessed as shown in Fig. 2. These results showed that participants in Arm C had more TB-specific unfavorable outcomes (confirmed failure and relapse cases) than participants in Arm B (primary endpoint) or Arm A (secondary endpoint; Figure 2A, B). Notably, participants in Arm A, deemed to have more severe disease, following 6 months of treatment, only experienced 7 relapses and one treatment failure out of 248 participants (3.2%). In Arm B, consisting of 132 participants with less severe disease randomized to the full 6 months of treatment, there were only 2 relapses (1.5%) and no failed treatment cases. This was only slightly lower than Arm A. In the four-month treatment arm, Arm C, amongst 141 participants there were 14 relapses and 3 treatment failures (12.1%).

Recurrent disease was assessed by mycobacterial interspersed repeat units-variable number of tandem repeats (MIRU-VNTR) genotyping and whole genome sequencing of the bacterial isolate from baseline compared to a sputum culture isolate obtained at the time of recurrence (Figure 2C). Amongst the 30 failed treatment and recurrence cases, 27 were observed from South Africa. Three failed treatment cases showed 0 SNP differences when comparing the baseline and final isolate, 21 relapses contained between 0-5 SNP differences when comparing baseline and recurrent isolates, and 3 participants were determined to have been reinfected based on between 237-1,265 SNP differences between their baseline and recurrent isolates (Figure 2C). For the three unfavorable outcomes from China only MIRU-VNTR genotyping data were available but both relapses and one treatment failure showed no change in MIRU-VNTR pattern. None of the observed SNPs in relapse strains were linked with acquisition of drug resistance.

### Primary and secondary endpoint analysis

For the primary endpoint, the Kaplan-Meier estimated proportion of treatment success was 98.4% in arm B and 86.7% in arm C (difference -11.7%, 95% CI, -18.2%, -5.3%). Most relapses occurred within the first year of treatment discontinuation (21/23, 91.3%) with an average time to relapse from treatment discontinuation of 205 days. We compared clinical, microbiological and radiological characteristics between patients in the two randomized Arms (B and C) to determine risk factors that correlated with poor outcomes. In univariate Cox proportional hazards analysis, assignment to the four-month early discontinuation arm (arm C) was the strongest predictor of unfavorable outcome with a hazard ratio (HR) of 7.99 (95% confidence interval CI: 1.84 to 34.7) (Table 2). Many features associated with cavities also showed significant p-values in this analysis including cavity volume at week 16 (p<0.001) and percent change in cavity volume from week 0 to week 16 (p=0.008). Total lesion glycolysis at week 4 (p=0.018) and 16 (p<0.001), change of total lesion glycolysis from week 0 to 16 (log ratio) (p=0.015), GeneXpert cycle threshold at baseline (p=0.009) and week 16 (p=0.012) were also significantly associated with unfavorable outcome. Surprisingly, however, hard lesion volume (volume within the range of Hounsfield Units (HU) of -100 to +200) did not correlate well with outcome, either as absolute values or as changes from baseline. In the multivariate analysis, assignment to Arm C remained the dominant risk factor (p=0.004) but total cavitary volume at week 16 (p=0.014) and total lesion glycolysis at week 16 (p=0.005) also remained significant. Adding participants from Arm A (who were stratified to all receive six months of treatment) into the analysis strengthened the association between 4-month treatment and unfavorable outcome, with total lesion glycolysis at week 16 and body mass index (BMI) at baseline also contributing to the multivariate model. Finally, to remove the large effect of treatment duration we performed an analysis of only those participants randomized to Arm C. In the multivariate model, total lesion glycolysis at week 16 and BMI at baseline remained the primary correlates of unfavorable outcome. We also analyzed this dataset using logistic regression and found similar results (Table S2).

### Baseline cavities reactivate during relapse

**Figure 3:**
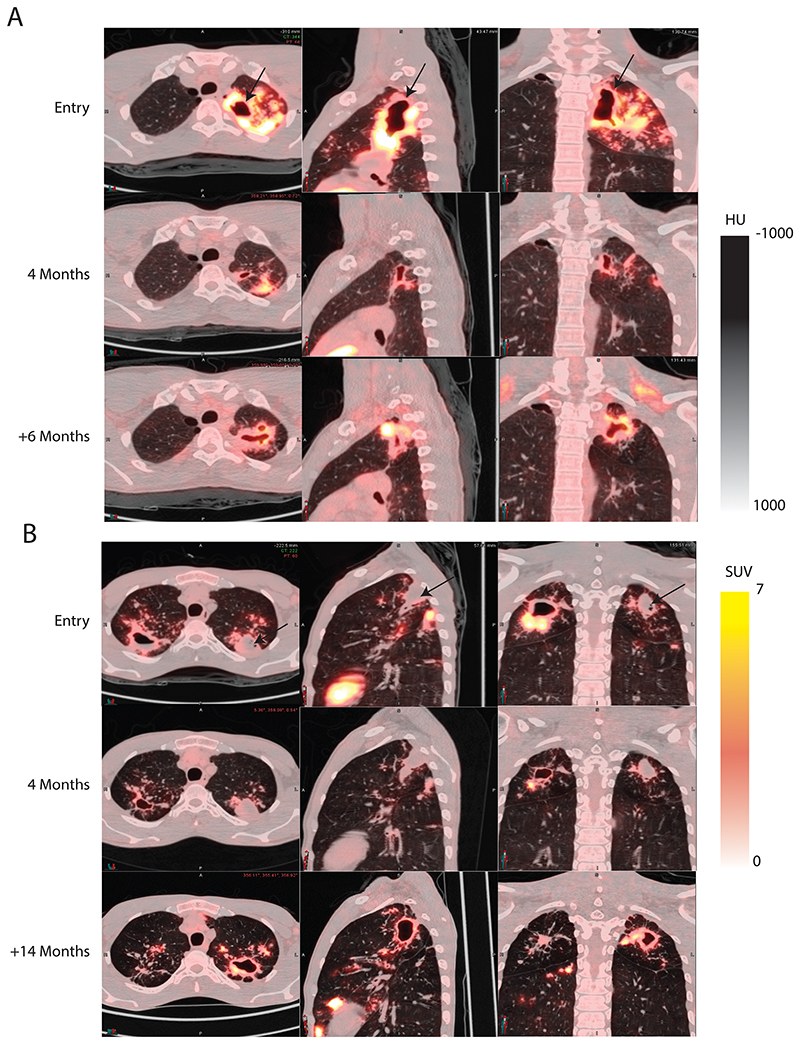
TB relapse involves baseline lung cavities. (**A)** Participant A19 in Arm A presented with a large left apical cavity on the PET/CT imaging scan(arrow top row), which appeared to have mostly resolved after four months of treatment. Six months after completing treatment the participant became sputum culture positive again with the same isolate and with a large left apical cavity in the same region of the lung. **(B)** Participant A9 presented with bilateral disease with a left upper “dirty” cavity (arrow on entry scans) with cold central necrotic material and only slight liquefaction. After four months of treatment the right apical cavity showed substantial response, whereas the cold left side lesion appeared less active. At 14 months from baseline the participant experienced relapse and became sputum culture positive with an identical isolate, and the necrotic left apical lesion appeared to be an active thick-walled cavity.

PET/CT imaging scans at the time of TB recurrence were obtained in 21 of the 23 study participants who experienced confirmed relapse (Figure 2C). All participants who relapsed had cavities at baseline on PET/CT imaging, although some were less than 2 ml in volume. In 17 of the 21 relapse PET/CT imaging scans, there was disease reactivation according to FDG-uptake on PET imaging with cavities that were strongly FDG-avid at the start of treatment and with reduced FDG uptake on treatment. Figure 3 shows two examples of relapse scans from Arm C participants. In the first example (Figure 3A), a large left apical thick-walled cavity which appeared to be in close contact with the posterior pleural surface mostly resolved after four months of treatment. The residual lesions at four months appeared unremarkable but there was a strong pleural connection in the axial and sagittal sections. Treatment was complete at this time per protocol. Six months after completing treatment the same cavity recrudesced, and the pleural connection extended. In the second example (Figure 3B), the participant presented at baseline with bilateral apical disease with a large right apical cavity with evident liquified caseum (lack of FDG labeling in the interior region of the cavity, best seen as an air-fluid level in the axial section). This “dirty” cavity phenomenon was also evident in a left apical cavity that appeared to have only begun the process of liquefaction. The left apical cavity also appeared to show more intimate pleural adhesion than the right side. Both cavities appeared to respond after four months of treatment although the left side lesion appeared to close off rather than resolve. Treatment was completed at four months, and upon relapse 14 months after therapy initiation, only the left cavity appeared to be the major sites of disease activity.

Two of the four participants who experienced confirmed relapse without reactivation of a cavitary lesion present at baseline were related and cohabitated during the study; the timing of their diagnoses was possibly consistent with reinfection with the same TB strain (Figure S2). In the third participant (A23), the relapse PET/CT imaging scan showed a small cavity at the original cavity site, but a new posterior lesion connected to that cavity by an inflamed bronchus was the main active lesion (Figure S3). As this scan was collected one year after treatment was discontinued, this new lesion was possibly seeded by the original cavity, a phenomenon we have previously reported (22). In the fourth participant (A27), there was no new lesion at the time of relapse and only sporadic low-level culture positivity, however, this scan was obtained five weeks after the participant restarted therapy making it difficult to know what the appearance was when the participant was diagnosed with relapse.

In four participants with failed treatment (defined as same strain recurrence by week 24), the active lesions observed on their third (and final) scans were all in the same location as their baseline lesions. On recurrence scans of all three cases of reinfection, active lesions were observed in areas of the lung that did not show involvement on prior scans and lesions present at baseline did not appear active at recurrence (see Figure S4 for an example).

### Post-hoc computational extraction and analysis of primary statistics

**Figure 4:**
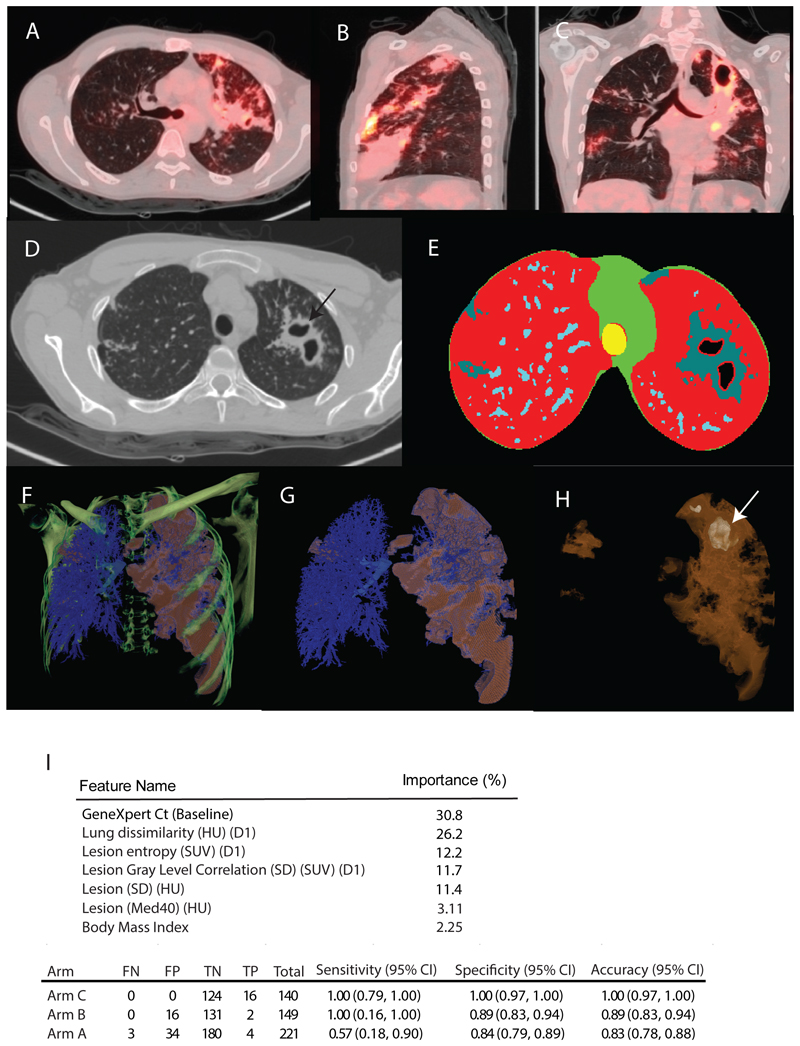
Scan segmentation and computational analysis. Shown are (**A**) axial (**B**) sagittal and (**C**) coronal views of PET/CT imaging scans of the lungs for participant A1 in Arm A at baseline. Displayed are CT scans viewed in the lung window and PET scans at a standardized uptake value (SUV)of 7. (**D**) Shown is a single slice through a CT scan for participant A1 indicating a lung cavity (arrow) and associated features. Scales for PET intensity and HU density are as in Figure 3.(**E**) Segmentations for the same CT slice as shown in panel **D** with low density normal lung voxels colored red, high density mediastinal and surface of thoracic cavity shown in green, trachea colored yellow, pulmonary vasculature shown in light blue and TB-associated lesional material shown in grey. (**F**) 3D multiplanar view of the same CT scan as in panel D showing CT bone density colored in green superimposed on a rendering of the 3D segmentations (low density normal lung removed for clarity), vasculature segmentation and major airways shown in blue, TB lesional material shown in brown. (**G**) 3D rendering of the vascular and lesion segmentations colored as in panel **F** with the primary CT data including the bones removed. (**H**) 3D rendering of only the lesion material with enhanced transparency to view the large left apical cavity (arrow). (**I**) Machine learning model for relapse prediction using Arm C PET/CT scans as the training set and Arms A and B PET/CT scans as the test set. Features include: GeneXpert cycle threshold (Ct) value at baseline; lung dissimilarity in Hounsfield units (HU), with dissimilarity in HU between adjacent voxels at a distance of 1 voxel (D1); lesion entropy (SUV) (D1), the entropy calculated across a lesion at a distance of 1 voxel; lesion gray level co-occurrence matrix (GLCM)(SD)(SUV)(D1): standard deviation of the Standardized Uptake Value (SUV) calculated across the lesion at a distance of 1 voxel; lesion (SD)(HU): standard deviation of HU within a lesion; lesion (MED40) (HU): lesion at 40^th^ percentile of HU value; l. BMI, Body Mass Index.

To gain deeper insights into the features linked to relapse, we created volumes of interest (segmentations) of the entire set of PET/CT scans using an enhanced version of our previously described automated computational segmentation method (23, 24). All scans were subjected to segmentation and then inspected manually (Figure 4A-H). High quality segmentations (eg Fig. 4E) were produced from the CT scans and then subjected to rigid co-registration based on the carina (the bifurcation point of the trachea) across visits. Sub-segmentations were also created for cavities (any interior airspace with contiguous lesion voxels around the air to capture cavity walls), hard lesion volume (HU values between -100 and +200) and soft lesion volume (abnormalities with HU density -500 to -101) (24). Each voxel within a segmented lesion was then used to compute various statistics of the material in the CT scan such as average, max and min HU (a full list of computed variables and an example are shown in Supplementary Materials and Methods). All scans were scaled to the same size and a common coordinate system was used within which volumes could be compared and those regions and their features associated with relapse then could be identified (Supplementary Materials and Methods).

Using the full set of both PET and CT statistics from participants in Arm C (at baseline), we trained a machine learning algorithm to discriminate relapsed participants from cured participants. Based on the top three performing configurations, the algorithm demonstrated excellent performance on the training set (Arm C), achieving zero false negatives and false positives in this cohort. When we evaluated the model’s performance on Arms A and B, where patient characteristics and treatment regimens differed from those in the training set, the model’s accuracy remained high, with scores of 0.83 (95% CI: 0.78, 0.88) and 0.89 (95% CI: 0.83, 0.94), respectively.

Because participants in Arms A and B were both treated for six months, the real performance using the model is better determined by considering the number of false positives. If these arms had been treated for four months we would have expected a relapse rate similar to that observed in Arm C (12.1%). Thus, among 132 subjects in Arm B, we would have expected 16 failures or relapses where the model determined 2 true relapses and 16 “false positives”. Likewise, amongst the 248 subjects enrolled in Arm A, the model captured 4 true positives and 34 “false positives” compared to the expected 30 failures/relapses expected with a 12.1% failure rate. Amongst the variables selected by the model as predictive (Fig. 4I), a texture feature called dissimilarity in HU density ranks highly. This feature value would be most highly associated with cavity walls where contrast in HU between interior air and the thick surrounding tissue would be most extreme. Several other variables relating to the relationship among HU density in lesion voxels were also suggestive of the importance of cavities in this relapse model. The relationship of PET SUV values across features contributed to two of the important variables predicting relapse. This suggests that it is not simply cavities, but rather cavities with specific FDG uptake related features that may predict relapse.

## Discussion

Our study set out to establish whether patients newly diagnosed with drug-sensitive pulmonary TB could be stratified into two cohorts; one that was and one that was not eligible for treatment shortening. Our criteria for stratification included TB disease severity at baseline by PET/CT imaging; early treatment response at one month and a GeneXpert cycle threshold minimum of 30 at four months. We based these criteria on a smaller cohort of patients with TB who had PET/CT imaging data available (25). These criteria were clearly unsuccessful at stratifying easier-to-treat versus harder-to-treat participants as our study was stopped early after a pre-specified interim analysis by our Data Safety Monitoring Board (DSMB). Our unfavorable outcome rates in the two six-month arms were 3.2% (Arm A) and 1.5% (Arm B), whereas the rate of unfavorable outcomes was 12.1% in the four-month treatment arm (Arm C). These outcomes were consistent with those observed in other studies that did not stratify participants by disease burden and treatment response, for example, the ReMoxTB trial, which had 2% relapse in the six-month control arm (and 3% retreatment) and 9-12% relapse in both four-month treatment arms (17, 20).

Baseline TB disease was different between the South African and Chinese cohorts, and this may be due to several factors including access to care, health-seeking behavior, genetics or baseline health status including body mass index. Large lung cavities are relatively uncommon and only 30 participants in the entire cohort had cavities larger than 30mL (Figure S5A). Amongst the unfavorable outcomes in Arm C that were not due to reinfection, the cavities that appeared to cause relapse ranged from 1-15mL in interior air volume (Figure S5B). Often these cavities appeared to be early in the process of liquefaction with evident caseum and thick walls and they frequently had intimate contact with the pleura.

Amongst the participants assigned to Arm A and designated harder to treat, baseline CT hard volume and PET activity accounted for the reason for relapse in 48% of the total participants in this arm, whereas large cavities only accounted for 13% (Figure S1). Hard CT volume represents a complex mixture of pathologies in TB disease ranging from cavity walls to larger areas of inflammation, fibrosis and dense airway infiltrates. Neither criterion was found to be associated with outcome in this study. Instead, the sixteen-week total glycolytic activity was significant in both univariate and multivariate analyses. This suggests that some lesions with major contributions to CT hard volume and total glycolytic activity are present at baseline but do not contribute to outcome prognosis – either because they are easier to treat or fibrotic. During the conduct of this study, we noted new lesions associated with cavities through bronchial spread (22). Such newly formed infiltrates are an obvious candidate for lesions that resolve quickly as they lack fibrotic structures that surround cavities and large nodules that limit drug access (26). These infiltrates are an important contributor to total glycolytic activity at baseline and appear to respond relatively quickly to treatment.

Likewise, CT hard volume and PET activity reductions at week 4 were not associated with relapse. In the end, arm A (participants meeting our definition of having higher risk for relapse) had only a very slight enrichment for relapse compared to Arm B (3.2% vs 1.5%) suggesting that our criteria based on the previous study did not identify patients at higher risk for relapse. The one-month timing of this evaluation was probably confounded by flares in glycolytic activity in many participants who otherwise responded well to treatment. These flares occurred in pre-existing lesions and were distinct from the appearance of new airway lesions described above. From the computationally segmented lungs, we observed that nearly one-third of all scanned participants (202 out of 648, 31%) experienced an increase in total glycolytic activity in their largest lesion at one month of treatment (Figure S6). This is likely a manifestation of a subclinical paradoxical reaction, which is common even in HIV-uninfected individuals (27). Paradoxical reactions are more frequently reported in extrapulmonary TB (28) although it may be that sequalae of such cases are more pronounced and it is therefore detected more frequently than in TB patients with primarily pulmonary disease.

Our study did establish unambiguously that lung cavities already present at baseline played a central role in relapse. This is consistent with the association seen with baseline cavity volume in our study and in many other studies (6–10). In addition, we noted that cavitary lesions with thick walls were present in nearly all relapse cases although they were also present in a large fraction of participants who did not relapse. The liquifying caseum within a thick-walled cavity was first noted as early as 1946 by Canetti et al. (29), who reported that the interior surface of the cavity was a primary site of rapid bacillary expansion and that as one proceeded towards the capsular lining the material was increasingly paucibacillary caseum. These “dirty” cavities are transient and ultimately result in a fibrous shell; Canetti proposed that such active processes are often over in a matter of days, a proposal supported by the rapidity of liquefaction in non-human primates (30). It seems possible that the application of chemotherapy during liquefaction interrupts the process to leave a caseum-adapted population of bacilli poised to reactivate when the selective pressure is removed.

To understand why some cavities are more difficult to treat than others we computationally segmented all PET/CT scans obtained during this study. This allowed us to compute detailed statistics for large numbers of lesions from each study participant at multiple time points and apply machine learning to predict features that were associated with relapse. The resulting model was successful at identifying lesions associated with relapse, particularly in Arm C patients used in training the model, but the model also performed well in Arm B participants, which the model had never seen. Performance of the model in the non-randomized Arm A patients was lower. The model could be improved by including more participants with extensive disease like that seen in Arm A. Evaluation of model performance in an independent population of TB patients is warranted. Three textural features account for almost 50% of the model’s predictive ability including lesion dissimilarity in radiodensity, lesion entropy in Standardized Uptake Value (SUV), and the correlation of SUV values in a grey layer correlation matrix. The pathophysiology resulting in these textural features is not intuitively obvious but suggests a heterogeneous distribution of values for SUV and HU across lesions that place a participant at higher risk for relapse with early treatment discontinuation. One unappreciated feature that could contribute to such heterogeneity is the extent of pleural adhesions of cavities to the chest wall. From the pre-chemotherapy era we know that pleural adhesions are very common in autopsies of people who died from TB. For example, in a review of 505 autopsies of patients with TB in Los Angeles from 1911-1950 as many as 85% displayed pleural adhesions (31). From the more modern era pleural adhesions have been described primarily as complications of surgical interventions to remove infected lungs (32). Interestingly, the main surgical complications appear to result from excess bleeding as these adhesions can recruit blood vessels from outside the thoracic cavity directly to the lesion.

Our study had some important limitations. Our criteria for more severe disease was based on a small cohort of study participants all of whom were treated with a full six months of therapy with a very small number of treatment failures and relapses. Evaluating TB patients with multiple PET/CT scans and intensive biomarker collection times is not translatable into typical TB care programs. Our study did not include individuals with HIV, an important comorbidity of TB and did not randomize almost half the total participants with radiologically extensive disease. Lastly, as the intervention specifically targeted lesion severity and bacterial load, this could have introduced bias in the analysis of lesion characteristics, thereby limiting the generalizability of the findings. Despite these limitations, our study revealed that a subset of patients had cavities with specific features that at baseline disease required more extensive treatment, but we have not yet identified a suitable biomarker to identify these patients. The majority of the relapses observed were in the South African cohort so we cannot rule out that the protocol was implemented with slight variations. We did determine that the overall extent of baseline disease was higher in South African patients and this may be responsible for the observed difference in relapse in that country.

Recent reanalysis of data from trials including the failed 4-month treatment shortening trials (17–19) has used some simple clinical features to stratify TB patients at baseline into three categories: low, moderate and high risk for relapse (33). Using this model, the low-risk group was shown to be non-inferior to standard of care after four months. The stratification used in this re-analysis relied on very simple clinical criteria such as smear grade, cavitation and body mass index and performed well. Prospective validation of a risk stratification system including these clinical features as well as an element related to cavity characteristics by CT may prove even more useful. Additional blood biomarkers of risk (such as cytokines and soluble indicators of cavitary disease) are being actively explored using data from this patient cohort and may enable additional risk stratification suitable for use in both new trials and in clinical care. This could reduce the size of future studies and allow more cost-effective comparisons of different treatment regimens. For future clinical trials of new agents and combinations, our study supports using clinical and radiological features to screen for participants who were “harder to treat” patients with a high likelihood of relapse as a prelude to a full Phase 3 enrolling all participants. This would allow a comparison of the treatment-shortening potential of multiple regimens in a Phase 2 trial prior to embarking on larger Phase 3 studies. Alternatively, the model could enable testing of only low risk TB patients to develop regimens that could result in much shorter regimens for patients with low-risk disease.

Aside from the utility of the model in our trial, our results suggest an explanation for the unusual distribution of relapses observed across all TB patients where 80% of patients were durably cured after 3-4 months of therapy. Specifically, relapses result from cavitary lesions with specific radiological features that are not present in most newly diagnosed patients with TB. The importance of this feature in non-human primates emerges independently in a completely different type of analysis of PET/CT data from treatment of TB infected marmosets in the companion paper in this issue (). This implies that the bacilli within these lesions are either fundamentally different from (e.g. sub-breakpoint MIC) or harder to treat than (e.g. due to drug penetration) other pathological manifestations. This finding will be helpful in focusing the search for new antitubercular medications active against these bacilli, allowing treatment shortening even for patients currently considered “high-risk”.

## Materials and Methods

### Study Design

The PredictTB clinical trial was a prospective, multicenter, non-inferiority, treatment-shortening, randomized trial of adult patients with newly diagnosed, drug sensitive pulmonary TB in South Africa and China (25). This study was registered at www.clinicaltrials.gov (NCT02821832).Briefly, adult patients with TB were enrolled at 5 sites in the Western Cape, South Africa (Stellenbosch University, University of Cape Town Lung Institute, University of Cape Town South African Tuberculosis Vaccine Initiative, Khayelitsha Site B, and TASK) and 5 sites in Henan Province, China (Henan Provincial Chest Hospital, Xinmi City Institute of Tuberculosis Prevention and Control, Kaifeng City Institute of Tuberculosis Prevention and Control, Zhongmu County Health and Epidemic Prevention Station, and Xinxiang City Institute of Tuberculosis Prevention and Control).

Inclusion criteria included adult patients with pulmonary TB who were GeneXpert MTB/RIF (*Mycobacterium tuberculosis* – rifampicin sensitivity test) positive for TB infection, were rifampin sensitive and had not yet started TB treatment. Exclusion criteria included extrapulmonary TB, pregnancy, HIV positivity, diabetes, isoniazid (INH) mono-resistance (determined by a local TB control program laboratory), or use of any immunosuppressive medications. Although diabetes is a common co-morbidity with TB, blood glucose is known to affect PET imaging quantitation and so patients with TB and diabetes were excluded(34). Likewise, HIV coinfected individuals are much more likely to fail TB treatment and so were excluded(35) to focus on TB-specific features associated with relapse these had to be exclude. Eligible participants signed informed consent and were started on standard of care fixed dose combination TB treatment with isoniazid, rifampicin, pyrazinamide, and ethambutol for 8 weeks, followed by isoniazid and rifampicin until treatment completion.

All participants received a thoracic FDG PET/CT imaging scan at baseline and at week 4. Participants in Arm B and C were all imaged at week 16 as well, and participants in Arm A were randomized to have a third scan at either week 16 or week 24. A possible fourth scan was done at the time of TB recurrence. A pre-specified scan reading algorithm was developed based on data from an earlier study (21) and two trained scan readers were randomly selected from a team of twelve physicians to read each scan. Scans with discrepant arm assignments between readers were read by a third reader. Participants were considered “low risk” if their baseline PET/CT scans did not show the following: total collapse of a single lung, a single cavity with a volume >30mL, CT hard volume (-100 to +100HU density) >200 mL or PET total glycolytic activity >1500 units. These criteria were based upon retrospective analysis of a previous cohort of 95 patients who had received similar scans by selecting for the minimum values that captured all relapses and failures in that study (21, 36). Participants exceeding any of these thresholds were considered high risk and were assigned to Arm A, receiving six months of standard treatment; participants who met all of these criteria were assigned to Arms B/C and started on standard treatment until they received a repeat PET/CT scan at 4 weeks. At the week 4 PET/CT scan, if participants failed to show a 20% decrease in cavitary air volume, showed an increase in CT hard volume of more than 10%, or an increase in PET glycolytic activity of more than 30% they were assigned to Arm A and not eligible for randomization. At week 16, participants still in Arms B/C were moved to Arm A if a GeneXpert cycle threshold was <30 (criterion changed from <28 after the initial 10 participants were randomized, due to more participants than expected *a priori* being stratified to Arm A at week 16) or if the participant had not taken at least 100/112 drug doses, based on data from the Medication Event Reminder Monitor, an electronic pill box (37, 38). Participants stratified to Arm A at baseline, week 4, or week 16 received standard of care TB treatment for 24 weeks. Participants who met all treatment shortening criteria at all 3 timepoints were randomized to Arm B and received standard of care TB treatment for 24 weeks or to Arm C and completed treatment at 16 weeks. All participants were followed to 72 weeks for final treatment outcomes. Patients who developed recurrent TB during follow-up had repeat PET/CT imaging scans, and their bacterial strains were isolated and whole genome sequencing was performed to distinguish relapse from reinfection.

Informed consent was obtained in the local language of the participant (English, Afrikaans, Xhosa, or Chinese). This study was reviewed and approved by the IRB/ethics committees of NIAID, Stellenbosch University, Faculty of Health Sciences University of Cape Town, South African Medicine Control Council, Henan Provincial Chest Hospital, and the Henan Center for Disease Control. The standing NIAID Data Safety Monitoring Board (DSMB) committee with three global TB experts added as *ad hoc* members provided oversight of the trial, meeting at least twice per year to review study data. Pre-specified stopping rules for inferiority of the treatment shortening arm and for study futility were provided to the DSMB. The first participant was enrolled in June 2017 in the Western Cape and in October 2017 in Henan Province. The last participant was enrolled in September 2020 in Henan and in March 2021 in the Western Cape, with follow-up completed 18 months later.

### Statistical methods

Cox proportional hazards models for the time to relapse/poor outcomes were fit in univariate and multivariate analyses to evaluate the associations between PET/CT characteristics, GeneXpert results, and host factors. Logistic regression analyses were fit for comparisons to Cox proportional hazards model. Confidence intervals for diagnostic accuracy, sensitivity and specificity were estimated using binomial exact confidence intervals. Analyses were conducted in R. Detailed power calculations for the study were reported previously (36) but in brief we had anticipated recruiting 155 subjects per arm, 140 to conclude non-inferiority based on a 97% treatment success rate and adjusting for loss to follow-up of 10% of participants. Our loss to follow-up was lower than anticipated so there were 141 evaluable participants in Arm C and 132 in Arm B.

## Supplementary Material

Figure S1

## Figures and Tables

**Table 1:** Baseline characteristics of participants by study arm and country. Xpert Ct: GeneXpert Ct. Cavities were defined as airspace >2 ml within the lung parenchyma with an interior HU value of <750 on the CT scan. Blank cells indicate that there were too few values to calculate *P*.Tot. vol., total volume.

		Total (N=517)*	ArmA (N=244)	ArmB (N=132)**	ArmC (N=141)**	South Africa (N=347)	China (N=170)*	P (CHN vs RSA)
sex	male N (%)	375 (72.5%)	188 (77.0%)	92 (69.7%)	95 (67.4%)	261 (75.2%)	133 (78.2%)	0.44
age	mean	35.3 (18-75)	36.8 (18-68)	35.2 (18-73)	32.8 (18-73)	34.1 (18-69)	37.7 (18-75)	0.01
	(range)							
BMI	mean (SD)	19.7 (3.0)	19.6 (3.0)	19.7 (2.7)	20.2 (3.2)	19.5 (3.1)	20.3 (2.7)	0.001
Prior TB	N(%)	92 (17.8%)	42 (17.2%)	23 (17.4%)	27 (19.1%)	90 (25.9%)	2 (1.2%)	<0.001
Smoker	N(%)	286 (55.3%)	134 (54.9%)	68 (51.5%)	84 (59.6%)	210 (60.5%)	76 (44.7%)	<0.001
Prior Smoking	N(%)	77 (14.9%)	47 (19.3%)	17 (12.9%)	13 (9.2%)	52 (15.0%)	25 (14.7%)	0.9
Duration	Mean (range)	17.4 (1.1-56.2)	18.6 (1.2-56.2)	16.5 (1.1-42.2)	16.0 (1.2-48.2)	17.1 (1.1-53.2)	18.2 (1.2-56.2)	0.34
Cavites								
none	N(%)	217 (41.7%)	59 (23.8%)	72 (54.5%)	86 (61.0%)	116 (33.4%)	101 (58.0%)	<0.001
1	N(%)	202 (38.8%)	109 (44.0%)	48 (36.4%)	45 (31.9%)	144 (41.5%)	58 (33.3%)	
2	N(%)	68 (13.1%)	53 (21.4%)	7 (5.3%)	8 (5.7%)	56 (16.1%)	12 (6.9%)	
3	N(%)	24 (3.4%)	18 (7.3%)	4 (3.0%)	2 (1.4%)	21 (6.1%)	3 (1.7%)	
>=4	N(%)	10 (1.9%)	9 (3.6%)	1 (0.8%)	0 (0.0%)	10 (2.9%)	0 (0.0%)	<0.001***
Tot. Vol. (mL)	mean (SD)	11.2 (19.8)	18.5 (25.6)	5.2 (8.3)	4.0 (6.8)	14 (21.8)	5.6 (13.2)	<0.001
Hard Lesion Vol.	mean (SD)	111.5 (92.1)	157.6 (106.2)	74.3 (50.2)	64.9 (44.4)	113.6 (95.4)	107.1 (85.2)	0.43
Total Lesion Glycolysis	mean (SD)	968.4 (830)	1311 (940.6)	717.3 (580.2)	601.1 (529.1)	1064 (814)	778.7 (831.4)	<0.001
Xpert Ct	mean (SD)	19.2 (5.2)	17.3 (4.5)	20.5 (5.0)	21.0 (5.5)	18.9 (5.0)	19.6 (5.6)	0.15
TTD MGIT	mean (SD)	7.9 (4.9)	6.9 (4.4)	9.2 (5.5)	8.8 (4.7)	7.6 (5.0)	8.9 (4.7)	<0.01

† P> 0.09 for all features between arms B and C.

‡ Comparison between participants with ≥3 lung cavities.

**Table 2:** Primary (arm B versus C) and secondary end point analyses. Univariate Cox PH analyses of variables associated with unfavorable outcomes combining arms B and C (first column), arms A + B + C (second column), or within arm C alone (third column). A hazards ratio (HR) > 1 indicates that increased values of the given characteristic correspond to faster times to relapse. Adjustments for multiplicity were not performed. Cl, confidence interval. Blank cells indicate that there were too few values to calculate P. Dash entries indicate the reference group.

	B+C Univariate Cox PH Model			A+B+C Univariate Cox PH Model			C only Univariate Cox PH Model		
Characteristic	HR^1^	95% CI^1^	p-value	HR^1^	95% CI^1^	p-value	HR^1^	95% CI^1^	p-value
**Arm**									
B	—	—		—	—		—	—	—
C	7.99	1.84, 34.7	0.006	8.19	1.89, 35.5	0.005	—	—	—
A	—	—	—	2.08	0.44, 9.81	0.4	—	—	—
**Country**									
CHN	—	—		—	—		—	—	
SA	3.2	0.93, 11.0	0.064	4.08	1.23, 13.6	0.022	2.98	0.86, 10.4	0.086
**Age**	1	0.96, 1.03	0.9	1.01	0.98, 1.03	0.6	1.01	0.97, 1.04	0.7
**Sex**									
Female	—	—		—	—		—	—	
Male	1.26	0.45, 3.51	0.7	1.32	0.53, 3.28	0.5	1.53	0.50, 4.71	0.5
**Prior TB episode**									
at least one previous TB episode not within the past 3 years	—	—		—	—		—	—	
otherwise	0.47	0.18, 1.25	0.13	0.49	0.22, 1.13	0.1	0.41	0.15, 1.11	0.08
**BMI**	0.84	0.70, 1.01	0.066	0.83	0.71,0.98	0.023	0.83	0.68, 1.01	0.062
**Weight**	0.95	0.91, 1.00	0.068	0.95	0.91, 0.99	0.023	0.94	0.90, 1.00	0.039
**Number of cavities week 0**	1.48	0.91, 2.41	0.12	1.33	0.96, 1.83	0.085	1.38	0.76, 2.51	0.3
**Cavity Volume week 0 (log2)**	1.07	0.98, 1.16	0.11	1.05	0.98, 1.12	0.2	1.06	0.97, 1.15	0.2
**Cavity Volume week 4 (log2)**	1.1	1.00, 1.21	0.063	1.05	0.97, 1.13	0.2	1.09	0.98, 1.21	0.11
**Cavity Volume week 16 (log2)**	1.19	1.08, 1.31	**<0.001**	1.11	1.02, 1.20	0.013	1.17	1.06, 1.30	**0.002**
**Cavity Volume change week 0 to 4 (log ratio)**	0.98	0.76, 1.26	0.9	0.94	0.75, 1.18	0.6	1	0.75, 1.35	>0.9
**Cavity Volume change week 0 to 16 (log ratio)**	1.11	0.97, 1.27	0.13	1.08	0.96, 1.22	0.2	1.09	0.96, 1.24	0.2
**Cavity Volume change week 0 to 4 (percent)**	0.54	0.17, 1.66	0.3	0.44	0.17, 1.16	0.1	0.61	0.18, 2.04	0.4
**Cavity Volume change week 0 to 16 (percent)**	1.2	1.05, 1.38	**0.008**	1.19	1.02, 1.39	0.024	1.16	1.01, 1.33	0.039
**Total hard volume week 0 (log2)**	1.45	0.93, 2.26	0.1	1.17	0.87, 1.59	0.3	1.4	0.88, 2.22	0.2
**Total hard volume week 4 (log2)**	1.33	0.91, 1.95	0.14	1.06	0.82, 1.38	0.6	1.35	0.90, 2.04	0.15
**Total hard volume week 16 (log2)**	1.16	0.82, 1.65	0.4	1.09	0.82, 1.45	0.6	1.18	0.82, 1.72	0.4
**Hard Volume change week 0 to 4 (log ratio)**	0.96	0.45, 2.05	>0.9	0.74	0.43, 1.27	0.3	1.14	0.53, 2.44	0.7
**Hard Volume change week 0 to 16 (log ratio)**	0.88	0.62, 1.24	0.5	0.93	0.66, 1.30	0.7	0.93	0.66, 1.32	0.7
**Hard Volume change week 0 to 4 (percent)**	0.91	0.16, 5.03	>0.9	0.75	0.22, 2.59	0.6	1.25	0.24, 6.50	0.8
**Hard Volume change week 0 to 16 (percent)**	0.42	0.10, 1.69	0.2	0.54	0.16, 1.83	0.3	0.48	0.12, 1.89	0.3
**Total lesion glycolysis week 0 (log2)**	1.34	0.95, 1.89	0.1	1.17	0.89, 1.52	0.3	1.32	0.93, 1.88	0.12
**Total lesion glycolysis week 4 (log2)**	1.5	1.07, 2.11	0.018	1.21	0.95, 1.52	0.12	1.58	1.09, 2.28	0.015
**Total lesion glycolysis week 16 (log2)**	1.76	1.27, 2.45	**<0.001**	1.41	1.11, 1.80	**0.005**	1.97	1.32, 2.94	**<0.001**
**Total lesion glycolysis change week 0 to 4 (log ratio)**	1.47	0.96, 2.26	0.074	1.28	0.85, 1.93	0.2	1.5	0.99, 2.28	0.057
**Total lesion glycolysis change week 0 to 16 (log ratio)**	1.46	1.07, 1.98	0.015	1.46	1.09, 1.96	0.012	1.47	1.08, 2.00	0.014
**Total lesion glycolysis change week 0 to 4 (percent)**	1.04	0.69, 1.57	0.8	0.98	0.59, 1.64	>0.9	1.04	0.71, 1.53	0.8
**Total lesion glycolysis change week 0 to 16 (percent)**	1.34	0.70, 2.58	0.4	1.43	0.79, 2.58	0.2	1.22	0.65, 2.32	0.5
**GeneXpert cycle threshold week 0**	0.87	0.79, 0.97	**0.009**	0.92	0.84, 1.00	0.057	0.89	0.81, 0.98	0.016
**GeneXpert cycle threshold week 16**	0.87	0.78, 0.97	0.012	1.01	0.95, 1.07	0.8	0.92	0.81, 1.05	0.2
**GeneXpert cycle threshold change week 0 to 16**	1.05	0.97, 1.15	0.2	1.05	0.98, 1.11	0.15	1.08	0.99, 1.18	0.095
**MGIT time to detection week 0 (log2)**	0.89	0.48, 1.65	0.7	1.02	0.61, 1.70	>0.9	1.07	0.55, 2.10	0.8
	**Multivariate Cox PH model**			**Mutivariate Cox PH model**			**Multivariate Cox PH model**		
**Arm**									
B	—	—	—	—	—		—	—	—
C	8.85	2.02, 38.8	**0.004**	10.6	2.39, 46.8	**0.002**	—	—	—
A	—	—	—	0.74	0.13, 4.25	0.7	—	—	—
**log2_CAVTOT_wk16**	1.14	1.03, 1.26	0.014	—	—	—	—	—	—
**log2 TLG wk16**	1.71	1.17, 2.48	**0.005**	1.82	1.32, 2.49	**<0.001**	1.98	1.34, 2.92	**<0.001**
**BMI**	—	—	—	0.83	0.71, 0.98	0.024	0.83	0.69, 0.99	0.042

## Data Availability

All data associated with this study are in the main text or supplementary materials. Study participant data can be made available upon completion of a clinical data sharing agreement with NIAID/NIH.

## Single Sentence Summary

Selecting patients with radiographically less-extensive tuberculosis for four-month treatment was inadequate to offset the increased risk of relapse, which occurred predominantly in thick-walled cavities already present at baseline.

## Accessible summary

We prospectively randomized participants with radiographically less-extensive tuberculosis disease (on FDG PET/CT) to receive either four or standard six months of chemotherapy. Those receiving four months of treatment had higher rates of relapse confirmed by whole genome sequencing of their bacterial isolates at baseline and recurrence. Repeat PET/CT scans showed relapse occurred in active cavities that were present at baseline. Post-hoc automated segmentation of PET/CT scans and machine learning analysis allowed accurate classification of participants that relapsed based on a combination of scan and clinical data. Efforts to shorten tuberculosis treatment duration should focus on rapidly killing bacteria in cavities.
